# External validation and clinical application of the predictive model for severe hypoglycemia

**DOI:** 10.3389/fendo.2022.1006470

**Published:** 2022-09-29

**Authors:** Jae-Seung Yun, Kyungdo Han, Soo-Yeon Choi, Seon-Ah Cha, Yu-Bae Ahn, Seung-Hyun Ko

**Affiliations:** ^1^ Division of Endocrinology and Metabolism, Department of Internal Medicine, St. Vincent’s Hospital, College of Medicine, The Catholic University of Korea, Suwon, South Korea; ^2^ Department of Statistics and Actuarial Science, Soongsil University, Seoul, South Korea

**Keywords:** hypoglycemia, type 2 diabetes, cohort studies, decision support system, clinical decision-making, validation study

## Abstract

**Objective:**

An internally validated, one-year risk prediction model for severe hypoglycemia (SH) in type 2 diabetes was evaluated in a general hospital setting to externally verify and validate its performance.

**Research design and methods:**

Between December 2017 to December 2019, 2,645 adult patients with type 2 diabetes who visited the diabetes center were enrolled. The receiver operating characteristics curve and Harrell C-statistics were compared to identify the discrimination of the model. The predicted and actual incidence of SH for one year in the development and validation cohorts were compared by ranking participants by deciles of predicted risk.

**Results:**

The concordance index was 0.878 in the external validation cohort. The sensitivity and specificity of the predictive model were 0.833 and 0.847, respectively. Based on the predicted risk, we stratified the groups into four categories: low (<0.05%), intermediate (0.05% to <0.5%), high (0.5% to <2.0%), and very high-risk group (≥2.0%). The actual annual incidence of SH gradually increased with the increased risk score level for the decile group (*P* for trend <0.001). The actual annual SH incidence significantly increased with increase in SH risk scores, which proportionately increased with age, duration of diabetes, glycated hemoglobin, and albuminuria and decreased with body mass index, renal function (*p* for trends <0.001 for all) in type 2 diabetes.

**Conclusion:**

On external validation, the novel one-year SH prediction model showed excellent discrimination in participants with type 2 diabetes and can effectively screen high-risk patients for SH, even in the general hospital setting.

## Introduction

According to data published by the International Diabetes Federation, the number of individuals with diabetes worldwide was estimated at 451 million in 2017 (1). Based on the continuing trend, the number is expected to increase to 693 million by 2045. The explosive increase in the prevalence of diabetes and its related complications has resulted in a greater need for effective diabetes care. Individualized medical evaluation and assessment of diabetes-related complications has been recently emphasized to achieve best practices in diabetes care (2). Comprehensive care for diabetes requires considerable resources and efforts (3). Therefore, screening is important to identify groups at high risk of diabetes complication for effective management with limited resources and time.

Hypoglycemia, especially severe hypoglycemia (SH), has a detrimental effect on quality of life and increases the risk of cardiovascular disease and mortality (4, 5). Similarly as with other complications, quantification of the risk of hypoglycemia and classification of high-risk patients can facilitate the prevention of SH (6). Previous studies have identified multiple risk factors that each contribute to SH in patients with type 2 diabetes and include antecedent hypoglycemia, hypoglycemia unawareness, older age, low body mass index (BMI), long diabetes duration, autonomic dysfunction, renal dysfunction, and strict glycemic control with insulin use (4). The currently recommended preventive strategies for SH are mainly based on a consideration of each risk factor that is causally related to SH events. However, there are limitations in the risk determination of SH with each individual factor. Therefore, a predictive model that can quantify and synthesize various risk factors is helpful for effective screening of patient groups at high risk for SH.

Despite their importance, there are few reports of prediction models for SH that can quantify and categorize the risk of hypoglycemia (7, 8). We have previously developed an 1-year predictive model for SH by using publicly available data and successfully validated the model with an internal validation dataset that was not independent of the original cohort (9). Moreover, because this model was developed from a nationwide population-based cohort, questions exist on whether it can be successfully applied in real-world practice, especially in the general hospital setting.

This study was conducted to verify the usefulness of a previously developed predictive model for SH in a hospital-based cohort which was independent of the original cohort.

## Materials and methods

### Brief description of the previously developed predictive risk model for severe hypoglycemia

A risk model for SH was previously developed from data obtained from the National Health Insurance Service (NHIS) health check-up database which comprises details of 1,173,820 individuals with type 2 diabetes (9). The outcome of SH was defined with ICD-10 codes for hypoglycemia in the claim records of the NHIS database. The first episode of an SH event per participant during the 1 year from the index date was included as the main outcome. We determined 14 candidate predictors which could be extracted from the NHIS database on the basis of both, significant statistical results from cohort analysis and clinically meaningful variables from previously published references. The final selected variables were: age, sex, smoking, alcohol consumption, body mass index (BMI), physical activity, insulin use, number of oral hypoglycemic agent (OHA) used, hypertension, chronic kidney disease (CKD), previous history of SH within the past 3 years, duration of diabetes (<5 and ≥5 years), fasting glucose level, and the Charlson Comorbidity Index (CCI) score. We assigned risk scores for the prediction of SH based on the hazard ratio for each risk factor in a Cox proportional hazards regression model (**Supplemental Table S1**). The risk-prediction model was translated into a risk-score nomogram. We undertook internal validation with 503,065 participants which were extracted from the same database in accordance with the Harrell’s bootstrap resampling method. The performance of the model was assessed with standard metrics, including c-statistics, chi-square statistics, and a calibration plot. The study methodology has been described previously (9).

### Study population

The diabetes complications registry of St. Vincent’s Hospital Diabetes Center contains accurate diabetes-related clinical information that has been collected through interview and chart review of the medical history. To verify the clinical effectiveness of the previous predictive model for SH, we enrolled patients with type 2 diabetes who visited at the University-affiliated Diabetes Center of St. Vincent’s Hospital from December 1, 2017 to December 31, 2019 and were followed up for 1 year from the baseline index date. The exclusion criteria were type 1 diabetes, age less than 30, and steroid use during the follow-up period of the analysis. This study was approved by the Institutional Review Board of the Catholic Medical Center of Korea (approval number:VC18FCSI0225) and was conducted in compliance with the principles of the Declaration of Helsinki, 1964.

### Definition of outcome

The primary outcome was SH during the follow-up period and was defined as hypoglycemia episodes requiring hospitalization or medical care in an emergency department (9). Medical staff in our clinic routinely checked with the patients on their experience of symptomatic and SH in accordance with the current guidelines for the clinical management of diabetes (2). The outcome of SH was confirmed by chart review of records from the emergency room. When an event of hypoglycemia in this study population was treated at another clinic or hospital, we requested the medical records to ascertain the treatment administered for hypoglycemia. Three participants experienced recurrent SH events during the follow-up period. In these cases, only the first episode of the SH event per participant was included as the main outcome.

### Exposures

Our clinical registry for diabetes complication contains patient information, including anthropometric, demographic, and social variables with information on the participant’s comorbidity, medication, and laboratory investigations. Heavy alcohol use was defined as alcohol consumption >30 g/day, and physical activity was defined as 30 minutes of moderate exercise ≥5 days per week or 20 minutes of vigorous exercise ≥3 days per week (similarly as in the original model). Hypertension was defined by systolic/diastolic blood pressure ≥140/90 mmHg or ongoing treatment with antihypertensive agents. The clinical diagnosis of established comorbidities was based on a review of the verified medical records. The CCI score was calculated with a previously defined formula (10) on the basis of comorbidity information in the registry and in electronic medical records. The estimated glomerular filtration rate (eGFR) was calculated with the Modification of Diet in Renal Disease Study Group Equation (11). Chronic kidney disease (CKD) was defined as the persistence of decreased eGFR of <60 ml/min/1.73 m^2^ (CKD stage ≥3) (12). After overnight fasting, the fasting plasma glucose was ascertained by an automated enzymatic method, and glycated hemoglobin (HbA1c) was assessed by high-performance liquid chromatography. Urinary albumin concentration was measured in the urine sample through enzyme immunoassay with immunoturbidimetry; the albumin-to-creatinine ratio was calculated by dividing the total albumin concentration by the creatinine concentration.

### Statistical analysis

Statistical analyses were conducted in R package version 3.6.3. Using descriptive statistics, continuous variables are presented as mean with standard deviation or median (IQR), and categorical data are shown as frequency (%). The independent Student’s *t*-test was used to compare differences between the means of continuous variable, and the chi-square test was used to determine the differences in the proportion of categorical variables. The incidence of SH was expressed as events per 1,000 patient-years. The risk score was calculated from risk scores and decile groups stratified by the score category in the previously developed risk-prediction model (**Supplemental Table S1**). As we registered only participants for whom all 14 variables were available, there was no participant with missing data. Discrimination was assessed by plotting the receiver operating characteristics (ROC) curve. Harrell C-statistics were calculated to test the predictive performance of the prediction model, and a value in the range of 0.80–0.90 was considered excellent. Both, observed and predicted risk of SH for 1 year were compared by groups (decile groups defined in the original cohort) as proposed in the original cohort. Tests for linear trends (*P* for trend) were calculated by treating the decile group as a continuous variable. To assess the contribution of the risk score for each variable, the mean contribution rate of each variable was calculated as the mean of the total risk score that was divided by the specific variable risk score of each participant. Thus, the contribution ratio refers to the mean percentage of a specific variable in the risk score of the total study sample or a specific decile group. Box- and bar-plots were used to visualize the distribution of each variable by using the risk-predictive model and mean contribution rates for the risk score of the main variables, respectively.

## Results

### Baseline characteristics of the cohort for external validation

**Figure 1:**
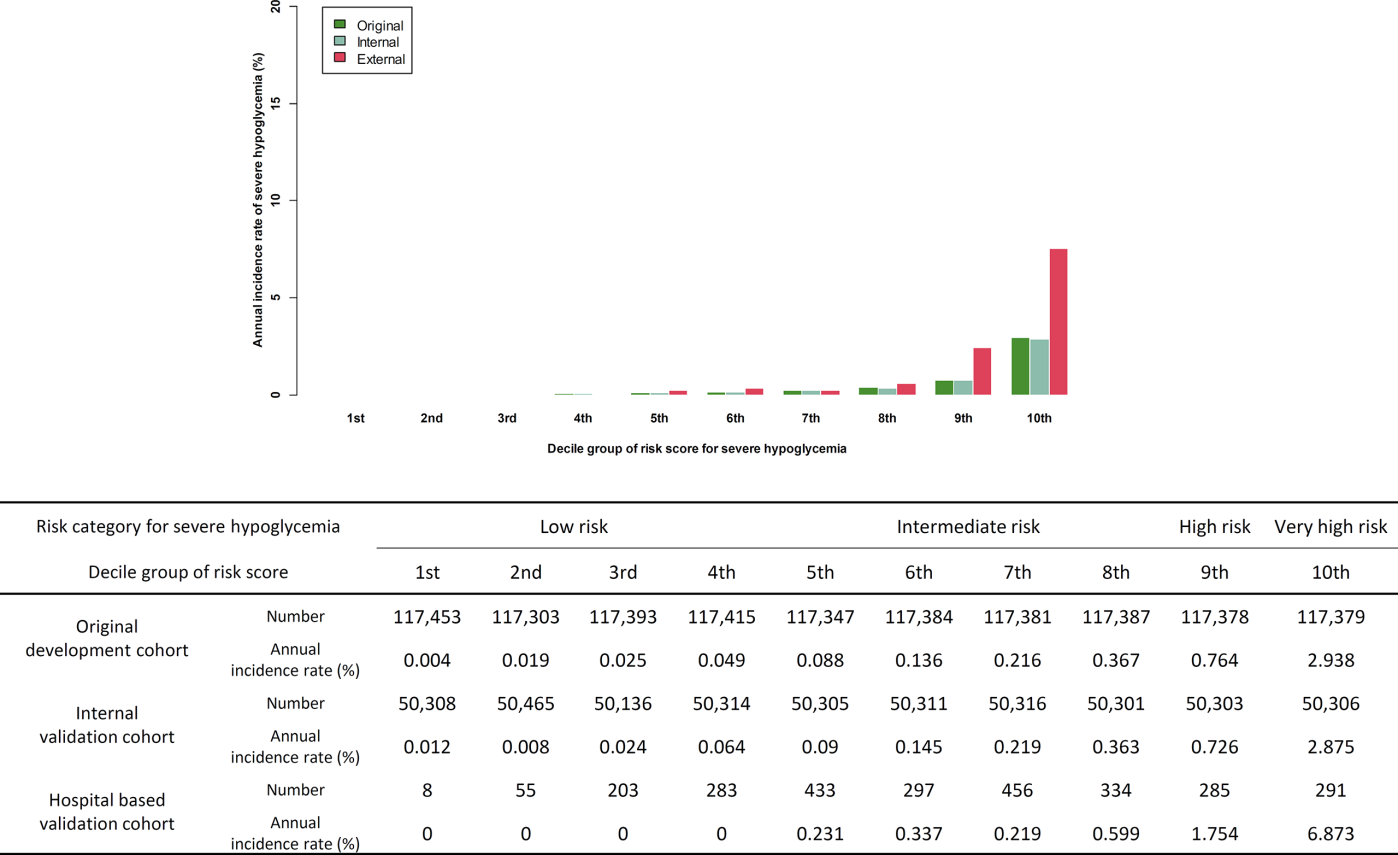
Comparison of predicted incidence rate per 1,000 person-year in development cohort and actual incidence rate in internal validation cohort and external validation cohort.

For external validation, a total of 2,645 subjects with type 2 diabetes were included from December 1, 2017 to December 31, 2019 in the study, after the exclusion of ineligible patients. At baseline, the mean age was 62.8 ± 12.2 years, the median duration of diabetes was 6.0 years, and 52.1% of the external validation cohort comprised male patients. The baseline eGFR was 88.6 ± 12.2 mL/min/1.73 m^2^. **Table 1** shows a comparison of baseline characteristics between the developmental and external validation cohorts. In the original prediction model, the number of each risk score decile group was almost equal (**Figure 1**). However, there were 8 (0.3%) participants of the first decile group in the hospital-based cohort, which was categorized by the assigned risk score from the original predictive model. The fifth decile risk-score group had the highest number of participants (n=433, 16.4%), and the 10th decile group had 297 (10.8%) participants.

**Table 1:** Baseline characteristics of original and external validation cohort.

	Original development cohort	External validation cohort
	(N=1,173,820)	(N=2,645)
Age (year)	57.9 ± 12.1	62.8 ± 12.2
Sex (male)	707,277 (60.2)	1,379 (52.1)
Diabetes duration (year)	N/A	6.0 (3.0-12.0)
BMI (kg/m^2^)	25.0 ± 3.3	25.2 ± 3.9
Number of SH history within recent 3 years		
0	1,166,019 (99.3)	2,618 (99.0)
1	6,525 (0.6)	24 (0.9)
>=	1,276 (0.1)	3 (0.1)
Insulin use (yes)	102,663 (8.7)	385 (14.6)
Number of oral hypoglycemic agent		
0	427,093 (36.3)	259 (9.8)
1	229,880 (19.6)	590 (22.3)
≥2	516,847 (44.1)	1,796 (67.9)
HbA1c (%)	N/A	7.2 ± 1.3
Fasting blood glucose (mmol/L)	7.9 ± 2.1	8.3 ± 3.8
eGFR (mL/min/1.73m2)	81.9 ± 21.2	88.6 ± 21.1
Albumin creatinine ratio (mg/g)	N/A	20.1 (10.0-54.7)

Data are presented as number (%), mean ± SD, or median (IQR).

BMI, body mass index; SH, severe hypoglycemia; eGFR, estimated glomerular filtration rate; N/A, Not Available.

### External validation of the predictive model for severe hypoglycemia

Of the 2,645 patients, 30 patients suffered novel-onset SH events. The incidence of SH was 11.3 per 1,000 patient-year, which is higher than the incidence rate in the original cohort (4.5 per 1,000 patient-year). Similarly as in the original cohort, participants who developed a new SH event were older, had lower BMI, comprised a higher ratio of female and insulin users, had a higher CCI score, and had a history of previous SH. The duration of diabetes and the HbA1c of the participants with SH was longer and higher, respectively, than in those without an SH event (**Supplemental Table S2**). The concordance index for SH was 0.878 (95% CI 0.865–0.891) in the external validation cohort. The sensitivity and specificity of the prediction model in the validation cohort was 0.833 and 0.847, respectively (**Supplemental Figure S1**). The actual SH event during 1 year in the 1st to 4th decile group was zero. With the increase in the level of the risk score for the decile group, the actual annual incidence of SH gradually increased (*P* for trend <0.001, **Figure 1**). We categorized the predicted risk groups as follows: low-risk group (annual predicted risk of SH <0.05%), intermediate-risk group (0.05% to less than 0.5%), high-risk group (0.5% to less than <2.0%), and very high-risk group (≥2.0%). Moreover, we pre-developed a simple version of the four-variable prediction model for SH, and the concordance index of this simplified version of the model was 0.793 (95% CI 0.777–0.808).

### Analysis of risk-score distribution by major variables

**Figure 2:**
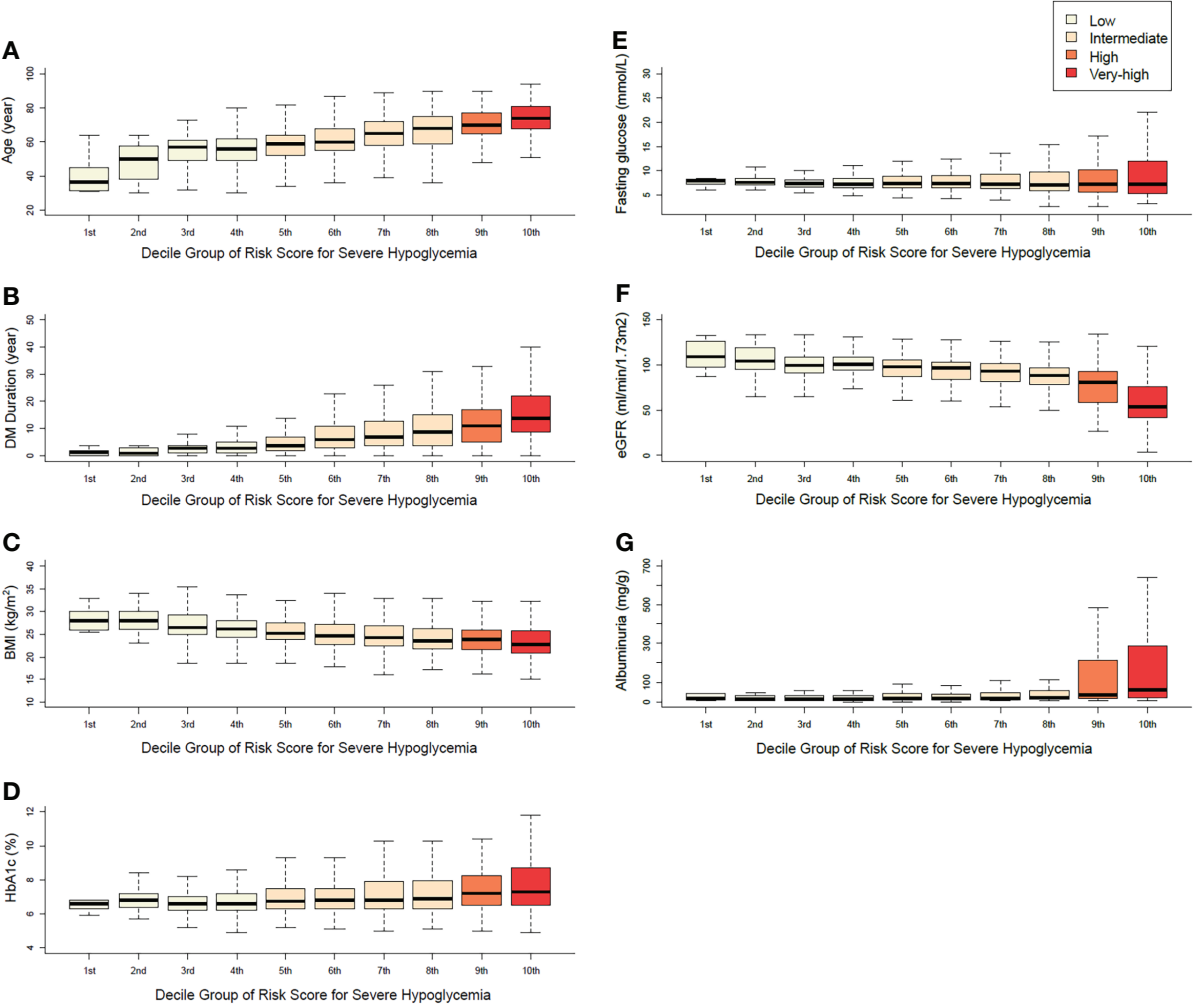
Distributions of the main variables in each decile group **(A)** age, **(B)** duration of diabetes, **(C)** body mass index, **(D)** HbA1c, **(E)** fasting plasma glucose, **(F)** eGFR, **(G)** albuminuria.

According to the distribution of the main variables in each decile group, the risk score increased as age, diabetes duration, fasting plasma glucose, HbA1c, and albuminuria tended to increase, whereas BMI and eGFR tended to decrease (*P* for trend <0.001). The range of fasting plasma glucose and HbA1c tended to be wider as the risk score for the groups increased (*P* for trend <0.001, **Figure 2**). The distribution of comorbidities and antidiabetic medication according to the risk-score decile groups is described in **Supplemental Table S3**. With the increase in the level of the risk score, the ratios of major comorbidities and insulin and sulfonylurea users exhibited a proportionally increasing trend.

## Discussion

External validation is an essential step in the development of any risk-prediction model before it is clinically applied. We validated previous our risk-prediction model of SH in a general hospital-based cohort. With the application of this predictive model for 2,645 participants, we found no actual incidence of SH in the 1st to 4th decile group (low-risk group), the incidence increased from the 5th decile group (intermediate risk group), and increased rapidly in the 9th (high-risk group) and 10th decile group (very-high risk group), with a similar slope pattern to that in the cohort used for the development or internal validation of the model. Despite the underestimation of SH risk in the high and very high-risk groups, the predictive model performed very well in terms of discrimination in the external validation samples. The main performance of this model was in the appropriate stratification of the high-risk group for SH; therefore, we focused on model discrimination over calibration to assess the performance of this model (13). This model can be used to easily and objectively categorize the risk of SH with quantifiable values. For example, Subject #325 in the external validation cohort was 79 years old, with a diabetes duration of 18 years. In this case, the medical staff can face confusion on whether to classify her into the high-risk group for SH due to her old age and long diabetes duration. However, this patient was not an insulin user, had no comorbidities or history of SH, exercised regularly for her health, and had a sufficiently high fasting glucose level (7.7 mmol/L) to not consider nocturnal hypoglycemia. Her total risk score was 70 points. According to the risk-prediction model, this participant belonged to the low-risk group. Eventually, she did not experience SH during the study period. Conversely, Subject #627, who was 41 years old, used three oral hypoglycemic agents, was being treated for AIDS, was heavy drinker, had a BMI of 17.5 kg/m^2^, and had a fasting glucose level of 4.7 mmol/L. His total risk score was 284 points, and this participant was assigned to the very high-risk group, and subsequently developed SH during the follow-up period. Moreover, we undertook to validate a four-variable simple model and the result showed good outcomes, but with relatively lower discriminatory power for predicting SH.

Previously, Karter et al. suggested a risk-prediction tool for SH to stratify the 1-year risk of SH with six factors (history of SH, prior visits to emergency room for any reason, insulin or sulfonylurea use, advance kidney disease, and age) (7). Their prediction model is simple, easy to apply, and showed good performance for risk prediction. When this was model was applied to our external validation cohort, only 15 subjects (0.6%) were classified into the high-risk group. Schroeder et al. developed a risk-prediction model for SH by using 16 variables (six variables for the simplified version), and successfully validated it in two external cohorts (8). Their model was rather complicated to use for risk calculation. When applied to this cohort, most of the study participants belonged to the low-risk group (84.8% and 9.0% of our cohort were in the 1^st^ and 2^nd^ quintile risk groups, respectively). These discrepancies could be due to differences in baseline cohort characteristics, exclusion criteria, racial differences, and definition of hypoglycemia between studies.

There were some differences in the characteristics between the data from the public NHIS cohort and that from the hospital-based cohort in our studies. Compared with those of the development cohort, the participants of the external validation cohort were generally in an advanced stage of diabetes, were older, had a higher rate of insulin use and multi-antihyperglycemic agents, and had higher fasting glucose levels than those of the original NHIS cohort. Because participants with advance renal disease in the external validation cohort usually transferred to nephrology clinic, the mean eGFR in the external validation cohort was better than in the development cohort. As the original development cohort for the predictive model from the public NHIS database comprised participants who can undergo regular medical check-ups, there is a possibility that patients with severe medical conditions were excluded from the development cohort.

The major strength of the NHIS data is the large sample size. However, diagnostic definitions were based on a medical claims database and, therefore, the misclassification of diagnoses is possible in a study that uses the publicly available NHIS data. In addition, due to the characteristics of the NHIS database, we could not sufficiently investigate the long-term glycemic status, diabetes duration, and albuminuria in the predictive model, and all of these have important clinical impacts on SH (14, 15). However, data on HbA1c and diabetes duration were fully available in the external validation cohort, and we could analyze these variables with other input variables of the previous risk model. The distribution of HbA1c according to the decile group is consistent with that of fasting plasma glucose. Although the risk score for the “fasting glucose” variable in the original score assignment was highest at the low fasting glucose level (<5.6 mmol/L, 46 points), the baseline mean fasting glucose level and HbA1c were highest and the distribution range of those variables were widest in the group at very high-risk for SH. In the NHIS data, the diabetes duration was only limited for use as <5 and ≥5 years. According to the distribution of diabetes duration by the risk-score group, diabetes duration increased proportionally with the risk score of the group. Although HbA1c and diabetes duration had limited value, the reason for the good performance of the original model can be explained based on other information such as age, fasting glucose, insulin use, and comorbidities that would cover the insufficiency of HbA1c and diabetes duration for the prediction of SH. In addition, all distributions of comorbidities and laboratory findings increased proportionally, which means that the overall composition of variables in this model was well balanced. Comprehensively, the limited diagnostic accuracy and insufficient information of HbA1c and diabetes duration did not significantly affect the performance of the predictive model.

According to the current guideline, the glycemic target should be adjusted to be less stringent in patients who are at high risk of potential hypoglycemia (2, 16). However, there is little evidence of the quantification and stratification of hypoglycemia risk. Prediction models can quantify the risk and support healthcare provider in assessing the risk and individualizing glycemic targets (17). For extensive application of the predictive model, several steps remain to be completed. It is necessary to verify the effectiveness of intervention that was categorized as a high risk in the predictive model (18). Possible interventions with high-risk group include frequent monitoring of glycemic parameters, lifestyle modification, reducing the intensity of the treatment regimen, and intensive patient education about hypoglycemia (6). We hope to undertake an interventional study that uses the predictive model to reinforce education for patients at high risk of hypoglycemia. These models can be used more easily by integration with a healthcare system or personal electronic health monitoring system. We created a formula that uses an interactive Web-based platform that automatically calculates the prediction score (http://md.koobian.com/sh/index.html). We believe that more effective treatment or prevention strategies for individuals in the high-risk group for SH will be possible with this predictive model, even in specialized diabetes care centers.

There were some limitations in our study. First, due to the low incidence rate, only a small number of outcomes occurred during the external validation study period. However, there were adequate significant trends of risk with this small number of outcomes, and the model showed good performance for prediction of SH. Second, because of the data security policy of the NHIS database, we could not directly compare the data between the external cohort and original cohort. The strengths of this study include the use of a registry-based cohort with variables that are clearly validated and access to a wide range of clinical predictors. We collected the information about SH from patient-interview questionnaires and chart review, without relying on the claim codes.

In conclusion, our predictive model developed from public database showed excellent discrimination and appropriately categorized high-risk groups for SH in patients with type 2 diabetes. We can use this model for screening and quantifying the risk of SH effectively, even in patients of general hospitals. Moreover, we can look forward to saving limited resources or efforts for proper care of hypoglycemia with this model. Further clinical interventional study for the prevention of hypoglycemia with this model is needed to verify the effectiveness of the predictive model of SH.

## Data availability statement

The raw data supporting the conclusions of this article will be made available by the authors, without undue reservation.

## Ethics statement

The studies involving human participants were reviewed and approved by the Institutional Review Board of the Catholic Medical Center of Korea (approval number:VC18FCSI0225). The patients/participants provided their written informed consent to participate in this study.

## Author contributions

J-SY wrote the manuscript and interpreted data. KH analyzed data. S-YC, S-AC contributed to discussions. Y-BA reviewed the manuscript. S-HK interpreted data, reviewed and edited the manuscript and contributed to discussions. All authors contributed to the article and approved the submitted version.

## Funding

This research was supported by a grant of the Korea Health Technology R&D Project through the Korea Health Industry Development Institute (KHIDI), funded by the Ministry of Health & Welfare, Republic of Korea (Grant number: HI18C0275). This research was supported by a grant (J-SY 2018F-5) from the Korean Diabetes Association.

## Conflict of interest

The authors declare that the research was conducted in the absence of any commercial or financial relationships that could be construed as a potential conflict of interest.

## Publisher’s note

All claims expressed in this article are solely those of the authors and do not necessarily represent those of their affiliated organizations, or those of the publisher, the editors and the reviewers. Any product that may be evaluated in this article, or claim that may be made by its manufacturer, is not guaranteed or endorsed by the publisher.
