# Supplementary material for: External validation and clinical application of the predictive model for severe hypoglycemia
Source: Front Endocrinol (Lausanne). 2022 Sep 29;13:1006470. doi: 10.3389/fendo.2022.1006470 (PMC9556834; doi:10.3389/fendo.2022.1006470)
Supplement: Supplementary file 1 [file DataSheet_1.docx]

**Supplemental Materials**

Supplemental Table S1 Risk scores for each risk factor category

| Variable | Categories and score | | | | | |
| --- | --- | --- | --- | --- | --- | --- |
| Age (years) | <40 | 40-64 | ≥65 |  |  |  |
|  | 0 | 20 | 49 |  |  |  |
| Sex | Male | Female |  |  |  |  |
|  | 0 | 3 |  |  |  |  |
| Current smoker | No | Current |  |  |  |  |
|  | 0 | 7 |  |  |  |  |
| Heavy drinker | No | Yes |  |  |  |  |
|  | 0 | 13 |  |  |  |  |
| BMI (kg/m^2^) | <18.5 | 18.5-22.9 | 23.0-24.9 | 25.0-29.9 | ≥30.0 |  |
|  | 59 | 29 | 11 | 1 | 0 |  |
| Regular exercise | Yes | No |  |  |  |  |
|  | 0 | 12 |  |  |  |  |
| Insulin use | No | Yes |  |  |  |  |
|  | 0 | 48 |  |  |  |  |
| Hypertension | No | Yes |  |  |  |  |
|  | 0 | 12 |  |  |  |  |
| Chronic kidney disease | No | Yes |  |  |  |  |
|  | 0 | 31 |  |  |  |  |
| Previous history of SH (n) | 0 | 1 | ≥2 |  |  |  |
|  | 0 | 73 | 100 |  |  |  |
| Diabetes duration (years) | <5 | ≥5 |  |  |  |  |
|  | 0 | 17 |  |  |  |  |
| Number of OHAs (n) | 0, 1 | 2 |  |  |  |  |
|  | 0 | 24 |  |  |  |  |
| Glucose level (mmol/L) | <5.6 | 5.6-6.9 | 7.0-8.2 | 8.3-9.9 | 10.0-11.6 | 11.7 |
|  | 46 | 15 | 0 | 5 | 9 | 22 |
| Charlson Comorbidity Index score (points) | 0 ,1 | 2 ,3 | 4, 5 | ≥6 |  |  |
|  | 0 | 31 | 52 | 74 |  |  |

BMI, body mass index; SH, severe hypoglycemia; OHA, oral hypoglycemic agent

Supplemental Table S2 Clinical characteristics between the groups with and without severe hypoglycemia event during the study period

|  | **Severe hypoglycemia (-)** | **Severe hypoglycemia (+)** | **P value** |
| --- | --- | --- | --- |
|  | **(N=2,615)** | **(N=30)** |  |
| Age (year) | 62.7 ± 12.2 | 74.8 ± 8.4 | < 0.001 |
| Sex (male) | 1368 (52.3) | 11 (36.7) | 0.128 |
| Presence of hypertension (%) | 1373 (52.5) | 21 (70.0) | 0.085 |
| Diabetes duration (year) | 8.2 ± 7.8 | 15.2 ± 10.3 | 0.001 |
| BMI (kg/m^2^) | 25.2 ± 3.9 | 23.1 ± 3.3 | 0.003 |
| Smoking (current) | 206 (7.9) | 3 (10.0) | 0.694 |
| Alcohol (heavy) | 111 (4.2) | 0 (0.0) | 0.415 |
| Activity (moderate or severe) | 986 (37.7) | 7 (23.3) | 0.212 |
| Number of SH event |  |  | < 0.001 |
| 0 | 2,593 (99.2) | 25 (83.3) |  |
| 1 | 20 (0.8) | 4 (13.3) |  |
| 2 | 2 (0.1) | 1 (3.3) |  |
| All Cardiovascular disease (yes) | 538 (20.6) | 10 (33.3) | 0.137 |
| Cancer (yes) | 304 (11.6) | 9 (30.0) | 0.005 |
| Chronic kidney disease (yes) | 269 (10.3) | 18 (60.0) | < 0.001 |
| Liver cirrhosis (yes) | 99 (3.8) | 1 (3.3) | 0.964 |
| COPD (yes) | 40 (1.5) | 0 (0.0) | N/A |
| Dementia (yes) | 56 (2.1) | 4 (13.3) | 0.001 |
| Insulin use (yes) | 375 (14.3) | 10 (33.3) | 0.008 |
| Number of oral hypoglycemic agent |  |  | 0.315 |
| 0 | 254 (9.7) | 5 (16.7) |  |
| 1 | 584 (22.3) | 6 (20.0) |  |
| 2 or more | 1,777 (67.9) | 19 (63.3) |  |
| HbA1c (%) | 7.2 ± 1.3 | 7.5 ± 1.5 | 0.260 |
| HbA1c (mmol/mol) | 8.3 ± 3.8 | 9.9 ± 7.3 | 0.226 |
| Fasting blood glucose (mmol/L) | 89.0 ± 20.8 | 57.7 ± 24.2 | < 0.001 |
| eGFR (mL/min/1.73 m^2^) | 4.1 ± 1.0 | 3.7 ± 1.0 | 0.062 |
| Albumin creatinine ratio (mg/g) | 155.2 ± 541.2 | 200.7 ± 386.9 | 0.722 |

Data are presented as number (%), mean ± SD, or median (IQR).

BMI, body mass index; SH, severe hypoglycemia; COPD, chronic obstructive pulmonary disease; eGFR, estimated glomerular filtration rate

Supplemental Table S3 The distribution of comorbidities and anti-hypoglycemic medication according to the risk score groups

| **SH Prediction Group** | **1st** | **2nd** | **3rd** | **4th** | **5th** | **6th** | **7th** | **8th** | **9th** | **10th** | ***P* for trend** |
| --- | --- | --- | --- | --- | --- | --- | --- | --- | --- | --- | --- |
|  | (N=8) | (N=55) | (N=203) | (N=283) | (N=433) | (N=297) | (N=456) | (N=334) | (N=287) | (N=293) |  |
| Coronary artery disease | 0 (0.0) | 4 (7.3) | 9 (4.4) | 16 (5.7) | 35 (8.1) | 23 (7.7) | 53 (11.6) | 55 (16.5) | 66 (23.0) | 63 (21.5) | <0.001 |
| Congestive heart failure | 0 (0.0) | 0 (0.0) | 3 (1.5) | 2 (0.7) | 5 (1.2) | 8 (2.7) | 12 (2.6) | 11 (3.3) | 18 (6.3) | 42 (14.3) | <0.001 |
| Cerebrovascular disease | 1 (12.5) | 1 (1.8) | 17 (8.4) | 14 (4.9) | 38 (8.8) | 42 (14.1) | 47 (10.3) | 52 (15.6) | 37 (12.9) | 52 (17.7) | <0.001 |
| Cancer | 0 (0.0) | 0 (0.0) | 3 (1.5) | 8 (2.8) | 25 (5.8) | 26 (8.8) | 62 (13.6) | 53 (15.9) | 49 (17.1) | 87 (29.7) | <0.001 |
| Chronic kidney disease | 0 (0.0) | 0 (0.0) | 0 (0.0) | 0 (0.0) | 3 (0.7) | 0 (0.0) | 4 (0.9) | 17 (5.1) | 74 (25.8) | 190 (64.8) | <0.001 |
| Liver cirrhosis | 0 (0.0) | 0 (0.0) | 0 (0.0) | 3 (1.1) | 7 (1.6) | 6 (2.0) | 17 (3.7) | 19 (5.7) | 25 (8.7) | 23 (7.8) | <0.001 |
| COPD | 0 (0.0) | 0 (0.0) | 0 (0.0) | 1 (0.4) | 4 (0.9) | 5 (1.7) | 7 (1.5) | 5 (1.5) | 7 (2.4) | 12 (4.1) | 0.009 |
| Dementia | 0 (0.0) | 0 (0.0) | 1 (0.5) | 1 (0.4) | 4 (0.9) | 1 (0.3) | 9 (2.0) | 13 (3.9) | 13 (4.5) | 18 (6.1) | <0.001 |
| Insulin | 0 (0.0) | 0 (0.0) | 0 (0.0) | 4 (1.4) | 12 (2.8) | 10 (3.4) | 43 (9.4) | 77 (23.1) | 105 (36.6) | 136 (46.4) | <0.001 |
| Sulfonylurea | 2 (25.0) | 12 (21.8) | 56 (27.6) | 88 (31.1) | 178 (41.1) | 155 (52.2) | 230 (50.4) | 179 (53.6) | 143 (49.8) | 159 (54.3) | <0.001 |
| Metformin | 5 (62.5) | 42 (76.4) | 146 (71.9) | 218 (77.0) | 331 (76.4) | 239 (80.5) | 368 (80.7) | 254 (76.0) | 225 (78.4) | 206 (70.3) | 0.045 |
| Meglitinide | 0 (0.0) | 0 (0.0) | 0 (0.0) | 0 (0.0) | 0 (0.0) | 1 (0.3) | 0 (0.0) | 3 (0.9) | 1 (0.3) | 7 (2.4) | <0.001 |
| DPP4 inhibitor | 0 (0.0) | 5 (9.1) | 45 (22.2) | 101 (35.7) | 206 (47.6) | 168 (56.6) | 269 (59.0) | 185 (55.4) | 159 (55.4) | 168 (57.3) | <0.001 |
| SGLT2 inhibitor | 1 (12.5) | 9 (16.4) | 22 (10.8) | 42 (14.8) | 63 (14.5) | 31 (10.4) | 43 (9.4) | 24 (7.2) | 16 (5.6) | 12 (4.1) | <0.001 |
| Thiazolidinedione | 0 (0.0) | 0 (0.0) | 0 (0.0) | 1 (0.4) | 2 (0.5) | 2 (0.7) | 9 (2.0) | 5 (1.5) | 5 (1.7) | 4 (1.4) | 0.219 |
| GLP1 receptor agonist | 1 (12.5) | 2 (3.6) | 6 (3.0) | 10 (3.5) | 16 (3.7) | 16 (5.4) | 16 (3.5) | 9 (2.7) | 5 (1.7) | 10 (3.4) | 0.468 |

SH, severe hypoglycemia; COPD, chronic obstructive pulmonary disease; DPP4, dipeptidyl peptidase 4; SGLT2, sodium glucose co-transporter 2; GLP1, glucagon like peptide 1


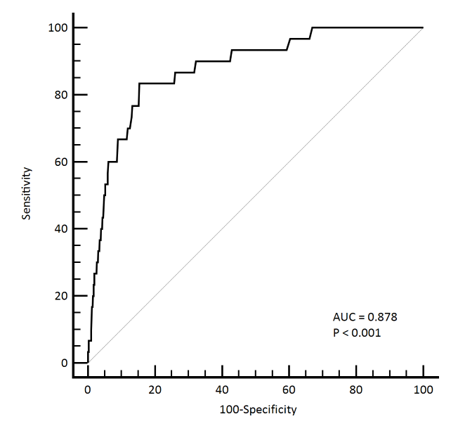


Supplemental Figure S1. Receiver operating characteristics curve for severe hypoglycemia in external validation cohort
